# INTERNATIONAL NORMALIZED RATIO AND ACTIVATED PARTIAL THROMBOPLASTIN TIME DO NOT PREDICT PLASMA TRANSFUSION IN LIVER TRANSPLANTATION

**DOI:** 10.1590/0102-6720202400061e1855

**Published:** 2025-01-13

**Authors:** David Silveira MARINHO, Joel Avancini ROCHA, Estela Regina Ramos FIGUEIRA, Claudia Regina FERNANDES, Rui Carlos DETSCH, José Huygens Parente GARCIA, Wellington ANDRAUS, Luiz Augusto Carneiro D’ALBUQUERQUE

**Affiliations:** 1 Department of Anesthesiology, Faculty of Medicine, Universidade Federal do Ceará – Fortaleza (CE), Brazil.; 2 Anesthesiology Unit, Hospital das Clinicas, Faculty of Medicine – Universidade de São Paulo, São Paulo (SP), Brazil.; 3 Department of Gastroenterology, Hospital das Clinicas, Faculty of Medicine, Universidade de São Paulo – São Paulo (SP), Brazil.; 4 Department of Surgery, Faculty of Medicine, Universidade Federal do Ceará – Fortaleza (CE), Brazil.

**Keywords:** International normalized ratio, Prothrombin time, Partial thromboplastin time, Blood transfusion, Liver transplantation, Coeficiente internacional normatizado, Tempo de protrombina, Tempo de tromboplastina parcial, Transfusão de sangue, Transplante de fígado

## Abstract

**BACKGROUND:**

Blood loss during liver transplantation (LT) remains a major concern associated with increased morbidity and reduced patient and graft survival. The high complexity of the procedure associated with the multifaceted origin of the bleeding urges early identification of high-risk patients and proper monitoring of hemostasis disorders in order to improve results. The accuracy of international normalized ratio (INR) and activated partial thromboplastin time (aPTT) to evaluate coagulation status in cirrhotic patients has been doubted.

**AIMS:**

The aim of this study was to investigate the applicability of these coagulation tests to indicate fresh frozen plasma transfusion in LT.

**METHODS:**

This retrospective cohort study analyzed 297 cirrhotic patients submitted to LT. INR and aPTT were measured preoperatively and in each surgical phase. Hemostatic blood components were transfused only for coagulopathy indication. Patients were divided according to intraoperative plasma transfusion into transfused and non-transfused groups. The accuracy of INR and aPTT to predict plasma transfusions was investigated. The alert values of INR and aPTT unassociated with coagulopathy in each phase of surgery were identified.

**RESULTS:**

Multivariate analysis showed that preoperative hematocrit (odds ratio [OR]=0.90, p<0.001), preoperative fibrinogen (OR=0.99, p<0.001), and absence of hepatocellular carcinoma (OR=3.57, p=0.004) were significant predictors of plasma transfusions.

**CONCLUSIONS:**

INR and aPTT demonstrated poor accuracy in predicting plasma transfusions, irrespective of the cutoff adopted, highlighting the need for a more comprehensive approach to guide hemostatic therapy in LT to improve the outcome.

## RESUMO

**RACIONAL:**

Sangramento durante o transplante de fígado continua sendo uma preocupação associada a maior morbidade e menor sobrevida do paciente e do enxerto. A alta complexidade do procedimento, associada à origem multifacetada do sangramento, reforça a necessidade de identificar precocemente pacientes de alto risco e monitorar adequadamente os distúrbios da hemostasia para melhorar os resultados. A precisão da razão normalizada internacional e do tempo de tromboplastina parcial ativada para avaliar o estado de coagulação em pacientes cirróticos tem sido questionada.

**OBJETIVOS:**

Investigar a aplicabilidade desses testes de coagulação para indicar a transfusão de plasma fresco congelado no transplante de fígado.

**MÉTODOS:**

Este estudo de coorte retrospectivo analisou 297 pacientes cirróticos submetidos ao transplante de figado. A razão normalizada internacional e o tempo de tromboplastina parcial ativada foram medidos no pré-operatório e em cada fase da cirurgia. Componentes sanguíneos hemostáticos foram transfundidos apenas por indicação de coagulopatia. Os pacientes foram divididos de acordo com a transfusão intraoperatória de plasma fresco congelado em grupos transfundidos e não transfundidos com plasma. Valores de alerta de razão normalizada internacional e do tempo de tromboplastina parcial ativada não associados à coagulopatia em cada fase da cirurgia foram identificados.

**RESULTADOS:**

A análise multivariada mostrou que hematócrito pré-operatório, fibrinogênio pré-operatório e ausência de carcinoma hepatocelular foram preditores significativos de transfusão de plasma fresco congelado.

**CONCLUSÕES:**

A razão normalizada internacional e o tempo de tromboplastina parcial ativada demonstraram baixa precisão para prever transfusões de plasma, salientando a necessidade de otimizacao do manejo hemostático perioperatorio para melhra dos resultados.

## INTRODUCTION

Despite all the advances in anesthesia and surgery, operative blood loss during liver transplantation (LT) remains a major concern, associated with increased morbidity and reduced patient and graft survival^9,19,21,23,26^. Given the complexity of the procedure, the multifaceted origin, and the volume of bleeding, it remains urgent to identify high-risk patients and properly monitor hemostasis disorders in order to improve outcomes^4,5,22^.

Regardless of their limitations in cirrhotic patients and the growing use of cell-based viscoelastic tests, plasma-based coagulation assays (PBCAs) remain the most used tests to monitor hemostatic competence during LT^3,23,24,29^. However, some studies have shown no correlation between PBCAs and bleeding parameters^3,7,17^. Although the international normalized ratio (INR) is an important marker of severity in acute and chronic liver diseases (CLDs), it does not seem to accurately reflect the complexity of hemostatic interactions in various clinical situations, such as CLD, trauma, hepatic surgery, and bleeding patients. Its ability to properly monitor perioperative hemostasis disorders has thus been questioned^14,27,29^.

In fact, the amount of bleeding may be largely influenced by other factors unrelated to the coagulation status — such as adhesions, portal hypertension, and surgical expertise — over which PBCAs have no predictive capacity^14,25^. In this study, we aimed to evaluate whether INR and activated partial thromboplastin time (aPTT) can predict fresh frozen plasma (FFP) transfusion in patients undergoing LT.

## METHODS

After approval by the ethics committee of the institution (number 083.08.10), the requirement for written informed consent was waived.

This is a retrospective cohort study of adult (>18 years old) cirrhotic patients who underwent deceased donor LT surgery over a 9-year period at Walter Cantídio Hospital, Universidade Federal do Ceará, Fortaleza (CE), Brazil.

### Exclusion criteria

Exclusion criteria included cases of combined transplants, repeated LT procedures, non-cirrhotic recipients, transplants for fulminant liver failure, patients on anticoagulants or platelet inhibitors, and patients with incomplete medical records.

### Surgical technique, anesthetic management, and intraoperative monitoring

Whole liver grafts were used from deceased brain-dead donors. Under general anesthesia, LT was performed using the piggyback technique. An arterial line was placed in the left radial artery, and a central line was inserted into the right internal jugular vein. The fluid regimen consisted of lactated Ringer’s solution (475 mL) mixed with 25 mL of human albumin 20%, infused at the anesthetist’s discretion based on hemodynamic and tissue perfusion variables. Blood loss was aspirated, anticoagulated immediately (30,000 IU of heparin in 1,000 mL saline), processed in a cell saver (Compact-Advanced^®^, Dideco, Mirandola, Italy), and reinfused into the patient when recovered.

### Laboratory and coagulation assays

Arterial samples were collected at four fixed time points (preoperatively within 6 h of surgery and at the end of each phase of the surgery: preanhepatic, anhepatic, and neohepatic) and stored in citrated tubes. The acid–base status and plasma levels of electrolytes, glucose, hemoglobin (Hb), and hematocrit (Ht), and standard coagulation tests (platelet count, fibrinogen by the Clauss method, aPTT, and INR) were monitored.

Platelet-poor plasma was obtained by centrifugation for 15 min at 3500 rpm and 25°C. Tests were performed on the Sysmex CA1500 (Sysmex Corporation^®^, Kobe, Japan). Thromborel^®^ S and Actin^®^ FS (Siemens Healthcare Diagnostics^®^, Marburg, Germany) were used to determine INR and aPTT, respectively. The International Sensitivity Index (ISI) values from Thromborel^®^ S were lot- and analyzer-specific, ranging from 0.93 to 1.12 across the study period. Coagulometer and reagents were used as per the manufacturer’s instructions. When blood samples were rendered incoagulable, the following values were assigned: INR=9.99 and aPTT=120 s. The turnaround time for the routinely measured tests was approximately 30 min.

### Coagulation monitoring

In addition to PBCAs, periodic visual assessments of the surgical field and communication with the surgeon were performed by the attending anesthesiologist to assess the presence of microvascular bleeding^12^.

### Requirements for functional hemostasis

A prophylactic infusion of epsilon-aminocaproic acid (EACA), 20 mg/kg/h, was maintained intraoperatively. The esophageal temperature was kept above 36°C, and ionic calcium levels were maintained above 1.1 mEq/L. Sodium bicarbonate was administered to keep bicarbonate levels around 20 mEq/L. Transfusions of salvaged blood units and packed red blood cells (PRBCs) were guided by laboratory results to maintain Ht above 25%. Intraoperative blood salvage was utilized in all cases, except for those involving neoplasms or contamination of the abdominal cavity.

### Diagnosis and management of hemostatic defects

Transfusion trigger: Regardless of the PBCA results, hemostatic blood components were not transfused unless microvascular bleeding was detected.

Diagnosis and management: Once microvascular bleeding was observed, the PBCA results were rank-ordered according to the deviation from values commonly considered adequate (INR <1.5, platelet count >50,000/mm^3^, and fibrinogen >100 mg/dL)^1^. Coagulopathy associated with an isolated elevation of INR was managed with coagulation factor replacement using FFP. When the coagulopathy was associated with an isolated low platelet count or fibrinogen levels, platelet concentrates or cryoprecipitate, respectively, was administered. In cases of multiple coagulation abnormalities, the most severe one was treated first. If the coagulopathy persisted after the first transfusion, other components were sequentially administered according to the severity of laboratory abnormalities. If coagulation tests indicated simultaneous deficiencies in coagulation factors and fibrinogen, FFP was initially transfused alone, as it contains both deficient components. If the FFP transfusion did not resolve the coagulopathy, a supplemental transfusion of cryoprecipitate was administered.

### Analysis of predictors of intraoperative fresh frozen plasma use during liver transplantation

Patients who received intraoperative FFP transfusions were categorized as the “FFP group” and those who did not as the “non-FFP group.” Demographic, surgical, laboratory, transfusion, and donor data were compared. Patients on the waiting list with hepatic tumors or other special conditions received exceptional points, leading to higher (corrected) Model for End-stage Liver Disease (MELD) scores^4^.

### Postoperative analysis

Survival at 1 week, 1 month, and 1 year, intensive care unit (ICU) stay duration until death or discharge to the wards, and early (<6 h) reoperations for microvascular bleeding were compared between the groups.

### Statistical data analysis

Descriptive statistical parameters were calculated for quantitative variables (mean and standard deviation [SD]) and qualitative variables (absolute and relative frequencies) for both groups. Baseline characteristics were compared using the χ^2^ test for categorical variables and Student’s t-test for normally distributed variables. Multiple logistic regression analysis was conducted to identify independent preoperative risk factors for FFP transfusions during LT. The Mann-Whitney test was used to compare ICU stay durations between the groups. Survival rates for both groups were calculated using Kaplan-Meier analysis, and a log-rank test was applied to assess survival differences. Receiver operating characteristic (ROC) curves were generated to evaluate the performance of PBCAs in predicting FFP transfusions during the entire LT and at each phase of surgery. For ROC curves specific to each phase of surgery, only patients who had not yet received an FFP transfusion at the time of blood sampling were included. The area under the ROC curve (AUC) was calculated. Cutoff values associated with the best sensitivity and specificity were identified. Diagnostic accuracy was categorized as fail (AUC≤0.6), poor (0.6<AUC≤0.7), fair (0.7<AUC≤0.8), good (0.8<AUC≤0.9), or excellent (0.9<AUC≤1.0). The significance level was set at p<0.05. Analyses were performed using Microsoft Excel 2003 (Microsoft Inc., Seattle, WA, USA), SPSS for Windows 15.0 (SPSS Inc., Chicago, IL, USA), and SAS 8.0 statistical software (SAS Institute Inc., Cary, NC, USA).

## RESULTS

### Patients’ characteristics

**Figure 1:**
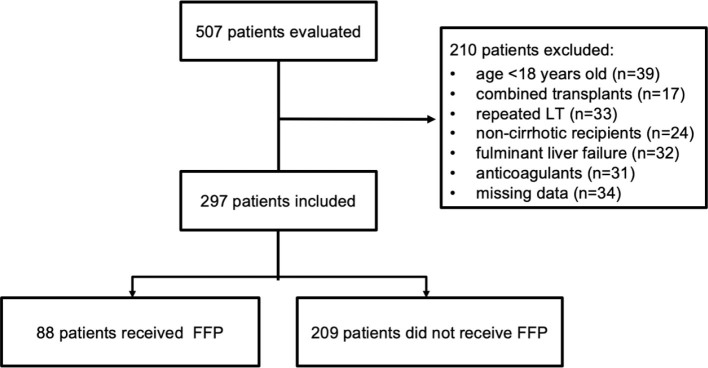
Flowchart.

FFP: fresh frozen plasma; LT: liver transplantation.

Of the 507 patients initially included in the study, 176 met the exclusion criteria, and 34 were excluded from the analysis due to incomplete data. Consequently, data from 297 patients were analyzed and they were divided into FFP group (n=88) and non-FFP group (n=209) (Figure 1).

Demographic, surgical, laboratory, transfusion, and donor data are shown in Table 1. Patients in the FFP group were younger and had significantly higher uncorrected MELD scores, lower aspartate aminotransferase values, a lower incidence of hepatocellular carcinoma, and worse preoperative values of Hb, Ht, and fibrinogen. Additionally, the FFP group had lower Hb levels by the end of surgery. This group also received more units of platelets and PRBCs per patient and showed statistically significantly higher transfusion rates for all blood products compared to the non-FFP group.

**Table 1:** Patients’ characteristics, and donor and transfusion data.

Variables	FFP group	Non-FFP group	p-value1
N	88	209	
Patient data			
Age: mean (SD), y	46 (12)	50 (12)	0.018
Male sex: %	67	77.5	0.059
Etiology: %			
C virus	28.4	27.8	0.644
Alcohol	25	25.4	
Cryptogenic	6.9	12	
B virus	10.2	10	
Other	29.5	24.8	
Uncorrected MELD: mean (SD)	19 (6)	17 (5)	0.009
Corrected MELD: mean (SD)	20 (6)	20 (5)	0.975
Hepatocellular carcinoma: %	8	25.4	<0.001
CIT: mean (SD), min	379 (114)	338 (97)	0.094
WIT: mean (SD), min	49 (13)	39 (10)	0.183
Esophageal T: mean (SD), °C			
End of preanhepatic phase	36.1 (0.2)	35.7 (0.6)	0.081
End of anhepatic phase	35.5 (0.1)	35.2 (0.2)	0.327
End of neohepatic phase	35.2 (0.5)	36 (0.8)	0.129
Hb: mean (SD), g/dL	10.8 (1.8)	11.8 (2.2)	<0.001
Ht: %	31.7 (5)	34.8 (6.2)	<0.001
Platelet count: mean (SD), × 109/L	85 (62)	100 (74)	0.084
Fibrinogen: mean (SD), mg/dL	162 (70)	207 (84)	<0.001
aPTT: mean (SD), s	44 (18.8)	36 (16.2)	0.228
INR: mean (SD)	1.9 (0.6)	1.6 (0.3)	0.223
Donor data			
Age: mean (SD), y	34 (2)	35 (2)	0.198
Sodium: mean (SD), mEq/L	153.4 (1.2)	153.9 (1.0)	0.614
AST: mean (SD), IU/L	91.3 (8.9)	108.9 (7.7)	0.042
ALT: mean (SD), IU/L	83.8 (5.7)	98.7 (4.9)	0.591
Transfusion data			
Platelet: mean (SD), units/patient	2.2 (3.6)	0.3 (1.4)	<0.001
Cryo: mean (SD), units/patient	0.3 (1.3)	0.3 (1.1)	0.887
FFP: mean (SD), units/patient	4.38 (1.9)	0	<0.001
PRBC: mean (SD), units/patient	1.5 (1.7)	0.4 (0.9)	<0.001
PRBC-transfused patients: %	56.8	21.5	<0.001
Platelet-transfused patients: %	31.5	6.2	<0.001
Cryo-transfused patients: %	4.5	3.3	<0.001
End of surgery-Hb: mean (SD), g/dL	8.1 (1.5)	9.5 (2.1)	<0.001
Platelet-, cryo-, or PRBC-transfused patients: %	71.6	28.7	<0.001

FFP group: patients who received at least one unit of FFP; Non-FFP group: patients who did not receive any unit of FFP during LT; SD: standard deviation; MELD: Model of End-stage Liver Disease; CIT: cold ischemia time; WIT: warm ischemia time; esophageal T: Esophageal temperature; Hb: hemoglobin; Ht: hematocrit; aPTT: activated partial thromboplastin time; INR: international normalized ratio; AST: aspartate aminotransferase; ALT: alanine transaminase; FFP: fresh frozen plasma; PRBC: packed red blood cells; LT: liver transplantation.

**Figure 2:**
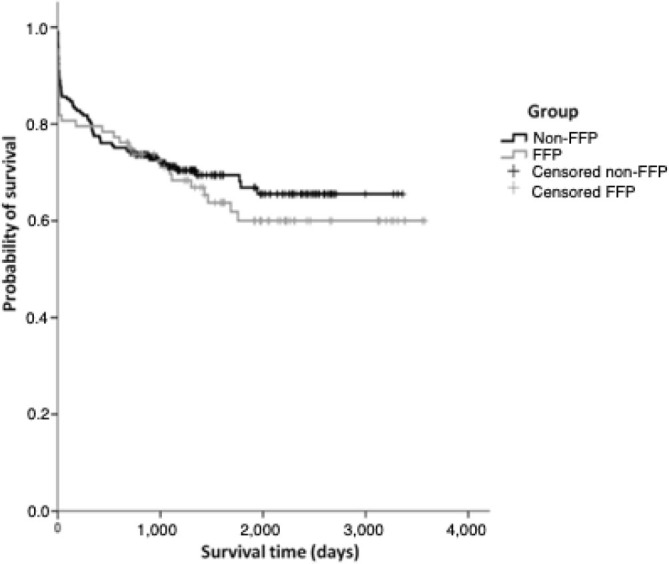
Kaplan-Meier curve for survival.

There was no statistically significant difference between the groups regarding ICU stay or the incidence of early reoperations for microvascular bleeding. Kaplan-Meier survival estimates coupled with a log-rank test showed a non-significant trend toward better long-term survival among patients who did not receive FFP transfusions (Figure 2 and Table 2).

FFP: fresh frozen plasma.

**Table 2:** Patients’ outcome according to the plasma-transfused group.

Outcomes	FFP group	Non-FFP group	p-value
No. of patients	88	209	
Early reoperations: %	8.5	10.2	0.723
ICU stay: mean (SD), days	3.6 (1.8)	4.3 (2.8)	0.082
Patient survival: %			
7 days	93.3	86.4	0.482
30 days	87.6	81.8	
1 year	77.5	79.5	

FFP group: patients who received at least one unit of FFP; Non-FFP group: patients who did not receive any unit of FFP during LT; Early reoperations: reoperations less than 6 h after LT for coagulopathic bleeding; ICU: intensive care unit; SD: standard deviation; FFP: fresh frozen plasma; LT: liver transplantation.

### Multivariate analysis to predict the use of fresh frozen plasma during liver transplantation

Although many variables exhibited some influence on the use of FFP during LT in the univariate analysis (Table 1), multivariate logistic regression analysis (Table 3) showed that preoperative Ht (odds ratio [OR]=0.90, p<0.001), preoperative fibrinogen (OR=0.99, p<0.001), and absence of hepatocellular carcinoma (OR=3.57, p=0.004) were the only statistically independent risk factors for FFP transfusions. Based on these results, it can be concluded that a 1% increase in preoperative Ht results in a 10% reduction in the chance of using FFP transfusions. Additionally, each 1 mg/dL increase in preoperative fibrinogen decreases the chance of receiving FFP transfusions by 1%. Finally, cirrhotic patients without hepatocellular carcinoma have a 3.57 times higher chance of requiring FFP transfusions compared to those prioritized for transplantation due to this neoplasm.

**Table 3:** Logistic regression analysis for predictors of fresh frozen plasma use during surgery.

Variables	OR	95%CI		p-value
		Inferior	Superior	
Preoperative Ht	0.90	0.85	0.95	<0.001
Preoperative fibrinogen	0.99	0.98	0.99	<0.001
Presence of hepatocellular carcinoma	3.57	1.50	8.48	0.004

OR: odds ratio; CI: confidence interval; Ht: hematocrit.

### Plasma-based coagulation assays to predict the need for fresh frozen plasma transfusions during liver transplantation

**Figure 3:**
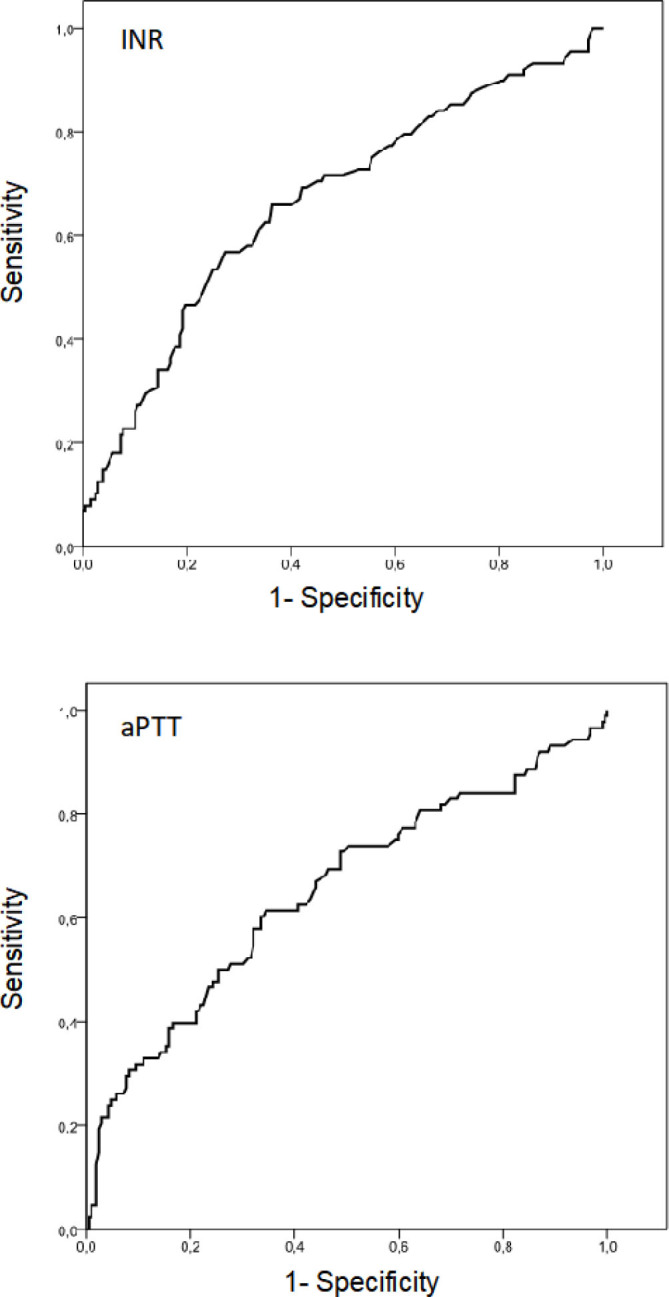
Receiver operating characteristic curves of preoperative plasma-based coagulation assays to predict fresh frozen plasma transfusions during liver transplantation.

Preoperative PBCAs, even at their best cutoffs, had poor accuracy in predicting the use of FFP during LT (AUC, sensitivity, and specificity values barely exceeded 60%). When evaluated phase-by-phase perioperatively, PBCAs also demonstrated poor accuracy (AUC, sensitivity, and specificity values did not exceed 70%) (Figure 3 and Table 4).

**Table 4:** Cutoff values for preoperative international normalized ratio and activated partial thromboplastin time to predict transfusions of fresh frozen plasma during liver transplantation.

Variables	Cutoff	Sensitivity (%)	Specificity (%)	PPV (%)	NPV (%)	AUC
Preoperative INR	1.605	65.9	61.2	41.7	81.0	0.636
Preoperative aPTT: s	34.75	61.4	59.3	38.8	78.5	0.603

INR: international normalized ratio; aPTT: activated partial thromboplastin time; PPV: positive predictive value; NPV: negative predictive value; AUC: area under the receiver operating characteristic curve.

Additionally, Table 5 summarizes the cutoff values associated with specificity as close as possible to 90%, identified from the ROC curves in Figure 3. The 90%-specificity cutoff values identified for samples collected in the preanhepatic, anhepatic, and neohepatic phases of LT were 2.14, 2.62, and 3.52 for INR and 50.5, 80.2, and 119.5 for aPTT, respectively.

**Table 5:** Best cutoffs and 90%-specificity of international normalized ratio and activated partial thromboplastin time, phase by phase (derived from the receiver operating characteristic curve curves in Figure 3).

Variables	Sampling time	Cutoff	Sensitivity (%)	Specificity (%)	PPV (%)	NPV (%)	AUC
INR	Preoperative	1.68	60	60.6	5	97.8	0.603
	Preanhepatic	2.18	71.6	66	47	84.7	0.688
	Anhepatic	3.75	65.9	66.5	45.3	82.2	0.682
INR-90	Preoperative	2.14	20	89.9	6.5	97	0.549
	Preanhepatic	2.62	42	90	63.8	78.7	0.66
	Anhepatic	3.52	47.7	90	66.7	80.3	0.658
aPTT: s	Preoperative	33.35	50	44.6	3.1	96.2	0.573
	Preanhepatic	45.85	64.3	60.6	14.5	94.2	0.624
	Anhepatic	83.30	67.2	60.9	32.1	87.1	0.641
aPTT-90: s	Preoperative	50.50	20	89.9	6.5	97.0	0.549
	Preanhepatic	80.20	32.1	90.0	25.0	92.7	0.611
	Anhepatic	119.5	54.7	79.4	42.2	86.4	0.630

INR: international normalized ratio; INR-90: INR cutoff at 90% of specificity; aPTT: activated partial thromboplastin time; aPTT-90: aPTT cutoff at 90% of specificity; preanhepatic: end of preanhepatic phase; anhepatic: end of anhepatic phase; PPV: positive predictive value; NPV: negative predictive value; AUC: area under the receiver operating characteristic curve; preoperative: preoperative phase less than 6 h.

## DISCUSSION

Transfusion practices in LT are characterized by wide variability, and differences in how coagulation abnormalities are managed with FFP and platelets may be even more significant than with the use of PRBCs^20^. Similar to other groups, our team employs a “wait-and-see” approach of rescue transfusion therapy, instead of prophylactic or preventive therapy, combined with maintaining hemostatic prerequisites, utilizing antifibrinolytics, using a cell saver, and relying on a skilled surgical team. Adopting such restrictive practices may help reduce FFP transfusion rates, as these may worsen LT perioperative results^11,18,19^.

Our overall reoperation rate for bleeding (9.1%) was in accordance with previous descriptions, although lower rates have been reported. These lower rates may reflect more effective transfusion protocols^11,18^. Long-term survival was longer in patients who did not receive FFP transfusions, although there was no significant difference between the groups in terms of survival and other postoperative outcomes. The adverse effects of FFP transfusions on perioperative results are not universal and seem to be dose-related. Therefore, our sample size may have been too small to detect such adverse effects, given the low transfusion rates presented. Additionally, potential differences in patient populations and criteria for group allocation may have contributed to these results.

In the FFP group, the transfusion of all blood products was more prevalent, and more units were transfused per patient, except for cryoprecipitate. These findings may reflect a more fragile hemostatic status or greater exposure to intraoperative challenges to hemostasis. Patients in this group had a lower incidence of hepatocellular carcinoma and higher uncorrected MELD scores^4^. The severity of liver disease is a well-known factor associated with transfusions during LT. Additionally, patients in this group had lower preoperative Hb and fibrinogen levels, both of which play critical roles in functional hemostasis^15,30^. The FFP group may have had a more fragile baseline hemostatic status, coupled with greater exposure to negative influences on hemostasis during LT, such as larger blood loss and dilution, which were not evaluated in this study^20^.

PBCA results, regardless of the sampling time, showed poor performance in predicting FFP transfusions during LT (Table 5). This inaccuracy has been previously described, although some of these studies used coagulation scores rather than PBCA results as predictors, and most evaluated blood loss or survival as the predicted outcomes^10,11^. Additionally, most previous studies restricted their analysis to preoperative coagulation tests and did not evaluate intraoperative measures. Furthermore, some of them employed conventional PBCA cutoffs, which were originally defined for non-cirrhotic patients.

Cirrhosis is commonly associated with clinical bleeding and abnormalities in PBCAs, making it tempting to infer a cause-and-effect relationship between these observations in both clinical and surgical scenarios^28,31^. However, prothrombin time (PT)/aPTT has several limitations in correlating with microvascular bleeding^29^. Despite these limitations, PBCAs are still the most commonly used tools for implementing goal-directed transfusion therapy. By identifying which part of coagulation is altered, selecting the appropriate blood component, and confirming therapeutic effectiveness, PBCAs may help reduce the use of blood products compared to relying solely on the visual assessment of the surgical field^8,32^.

The multivariate analysis supported the poor predictive performance of PBCAs, demonstrating that the only independent predictors of FFP transfusions during LT were preoperative Ht, preoperative fibrinogen levels, and the absence of hepatocellular carcinoma. According to this and other studies, these predictors can identify a group of LT patients who are more severely ill and more likely to receive transfusions^8,16,32^.

Global coagulation tests have shown good negative predictive values for bleeding in the early postoperative period. However, their isolated use to predict bleeding has consistently shown poor positive predictive values. To overcome this limitation, the authors have recommended correlating pathological results with clinically significant bleeding. This highlights the importance of clinical evaluation of microvascular bleeding over isolated coagulation test results, especially when triggering transfusions. As a result, microvascular bleeding has become increasingly accepted as a formal indication for FFP transfusions, to the detriment of relying solely on PBCA results, and has been included as a key step in hemostasis protocols^2,6^. This approach adds a layer of safety against laboratory errors and avoids potentially unnecessary transfusions, although a certain degree of subjectivity is inherent in most clinical transfusion studies^6,10,13^.

Additionally, coagulation assays have become important in guiding when not to transfuse FFP. Guidelines from the American Society of Anesthesiology regarding perioperative blood transfusions state that the transfusion of FFP is not indicated if PT, INR, and aPTT are normal^2^. In line with this recommendation, we evaluated critical PBCA values in patients who did not receive FFP transfusions. For this purpose, we arbitrarily chose cutoff levels that maximized specificity (90%), with the known trade-off of reduced sensitivity and overall prediction accuracy. In our scenario, for each phase of surgery, we found that 90% of patients who did not receive FFP transfusions had results below the calculated cutoffs. Clinically, this suggests that only 10% of patients who did not receive FFP transfusions reached the cutoffs shown in Table 5. Thus, patients with a dry surgical field who exceed these 90%-specificity cutoffs deserve frequent and meticulous monitoring to identify coagulopathy as it develops.

### Limitations of the study

Our study has some limitations. First, it is a single-center, non-randomized retrospective cohort study. However, controlled studies in patients with significant blood loss are difficult to conduct, as it would be unethical to include a control group that receives no hemostatic therapy. Second, the multifactorial etiology of coagulopathy during LT complicates the task of isolating all factors influencing PBCA abnormalities. Third, similar to other transfusion decision-making studies, the diagnosis of microvascular bleeding may involve some degree of subjectivity, and time lags may have hindered the real-time assessment of coagulation status.

Despite these limitations, we emphasize that this is a large investigation to assess the correlation between PBCA and FFP transfusions during LT, potential cutoffs, and their accuracy in such prediction. The proposal of high-specificity cutoff values for PBCAs in cirrhotic patients undergoing LT is also intriguing.

## CONCLUSIONS

Our results showed that the significant predictors for FFP transfusions were preoperative Ht, preoperative fibrinogen, and the absence of hepatocellular carcinoma. FFP transfusions did not influence survival, ICU stay, or the incidence of reoperations. PBCAs, regardless of the cutoffs adopted or the time of sampling during LT, had a poor correlation with intraoperative FFP transfusions. Therefore, in LT centers where global hemostasis tests are unavailable, PBCA values should not be used to trigger FFP transfusions but may be used as a reference for when not to transfuse FFP.
